# The Involvement of Cysteine-X-Cysteine Motif Chemokine Receptors in Skin Homeostasis and the Pathogenesis of Allergic Contact Dermatitis and Psoriasis

**DOI:** 10.3390/ijms25021005

**Published:** 2024-01-13

**Authors:** Wenjie Liu

**Affiliations:** State Key Laboratory of Cellular Stress Biology, School of Pharmaceutical Sciences, Xiamen University, Xiamen 361102, China; wjliu@xmu.edu.cn; Tel.: +86-592-2182453

**Keywords:** allergic contact dermatitis (ACD), psoriasis, cysteine-X-cysteine motif chemokine receptor, CXCRs, T cells

## Abstract

Members of the C-X-C motif chemokine receptor (CXCR) superfamily play central roles in initiating the innate immune response in mammalian cells by orchestrating selective cell migration and immune cell activation. With its multilayered structure, the skin, which is the largest organ in the body, performs a crucial defense function, protecting the human body from harmful environmental threats and pathogens. CXCRs contribute to primary immunological defense; these receptors are differentially expressed by different types of skin cells and act as key players in initiating downstream innate immune responses. While the initiation of inflammatory responses by CXCRs is essential for pathogen elimination and tissue healing, overactivation of these receptors can enhance T-cell-mediated autoimmune responses, resulting in excessive inflammation and the development of several skin disorders, including psoriasis, atopic dermatitis, allergic contact dermatitis, vitiligo, autoimmune diseases, and skin cancers. In summary, CXCRs serve as critical links that connect innate immunity and adaptive immunity. In this article, we present the current knowledge about the functions of CXCRs in the homeostasis function of the skin and their contributions to the pathogenesis of allergic contact dermatitis and psoriasis. Furthermore, we will examine the research progress and efficacy of therapeutic approaches that target CXCRs.

## 1. Introduction

The human skin is the largest organ in the body, and it functions as a protective barrier; the skin is continuously exposed to pathogens, environmental antigens, and commensal microorganisms, leading to the development of a robust immune defense system in the skin. Upon infection, allergen exposure, or injury, the innate immune system of the skin produces cytokines that bind to their corresponding receptors to enhance the defensive capabilities of the skin and actively shape and modulate adaptive immune responses in the skin; these responses are essential for maintaining the integrity of the skin barrier and promoting the overall health of the organism. Chemokine receptors are G protein-coupled receptors (GPCRs) that relay signals from the external environment into the internal cellular milieu via the activation of intracellular signaling pathways [1]. Upon exposure to foreign antigens or injury, chemokine receptors are activated by interacting with specific chemokines, which are released by various cells in the affected tissue. When chemokines bind to their receptors on the surface of a cell, the receptors undergo a conformational change. This conformational change activates G proteins, which are located on the intracellular side of the receptor. Once activated, the G protein dissociates into its subunits (α, β, and γ), leading to the activation of downstream signaling pathways [2]. This activation triggers a cascade of intracellular events, such as the activation of enzymes, the production of secondary messengers, and the modulation of gene expression [3]. Ultimately, these signaling pathways regulate immune cell migration, inflammation, and other immune responses that are involved in fighting infection or promoting tissue repair [1].

Mammals have four major classes of highly conserved and distinct chemokine receptors, including C-C chemokine receptors (CCRs), C-X3-C chemokine receptors (CX3CRs), X-C chemokine receptors (XCRs), and C-X-C chemokine receptors (CXCRs). Among the different classes of chemokine receptors, CXCRs have been extensively studied [4,5,6,7,8]. Several researchers initially discovered and characterized CXCRs. The first C-X-C chemokine receptor, namely CXCR1, was identified through a combination of molecular cloning and functional assays; this receptor was found to be a high-affinity receptor for interleukin-8 (IL-8 or CXCL8). Later, another C-X-C chemokine receptor, namely, CXCR2, was identified using similar molecular biology techniques. These early discoveries laid the foundation for further research on CXCR family members and their involvement in immune responses and diseases. The discovery of CXCR1 and CXCR2 provided evidence that these receptors perform central functions in mediating the recruitment and activation of immune cells during inflammation and immune responses. This breakthrough discovery confirmed the importance of chemokine receptors in immune cell trafficking and paved the way for further research into their functions, signaling pathways, and involvement in multiple physiological and pathological processes.

The unique multilayered structure of the skin plays a crucial role in maintaining skin homeostasis and orchestrating immune responses. The epidermis, which is the outermost layer of the skin, provides a protective shield against pathogens, UV radiation, and chemical substances. Additionally, the epidermis contains specialized immune cells, such as Langerhans cells, that detect and respond to foreign substances. The dermis, which is located beneath the epidermis, contains blood vessels, lymphatic vessels, and immune cells that help regulate skin regeneration and immune responses. When chemokines bind to CXCRs on resident cells [9], such as keratinocyte dendritic cells, a series of signaling events that lead to immune activation is triggered. This immune activation results in the synthesis of proinflammatory cytokines, such as TNF and interleukins, which are involved in the initiation and progression of inflammation within the skin. A proper interaction between immune cells, including T cells and dendritic cells, within skin layers is essential for facilitating immune surveillance, maintaining skin homeostasis, and orchestrating immune responses [10].

CXCR activation performs an essential function in immune cell recruitment and activation during inflammation, facilitating immune responses against infection, allergens, or injury. On the other hand, excessive or dysregulated CXCR activation can contribute to the persistence of chronic inflammation and the tissue damage that is observed in autoimmune and inflammatory diseases, including psoriasis [11,12], atopic dermatitis (AD) [13], allergic contact dermatitis (ACD) [14,15], vitiligo [16,17], and skin cancers [18]. Therefore, maintaining a balance of CXCR activation is essential for exploiting its beneficial effects while preventing potential detrimental consequences. Here, we will review how abnormal activation of CXCRs leads to ACD and psoriasis, as well as the latest progress in CXCR-targeted treatment of these skin diseases.

## 2. CXCR-Mediated Cellular Communication

CXCRs are dynamic, seven-transmembrane-domain proteins that modulate a range of intracellular signaling cascades in reaction to hormones, neurotransmitters, ions, photons, odorants, and other stimuli. As such, they perform crucial functions in physiology and disease and are desirable targets for pharmaceutical intervention.

### 2.1. The Architecture of CXCRs and Their Corresponding Ligands

**Figure 1:**
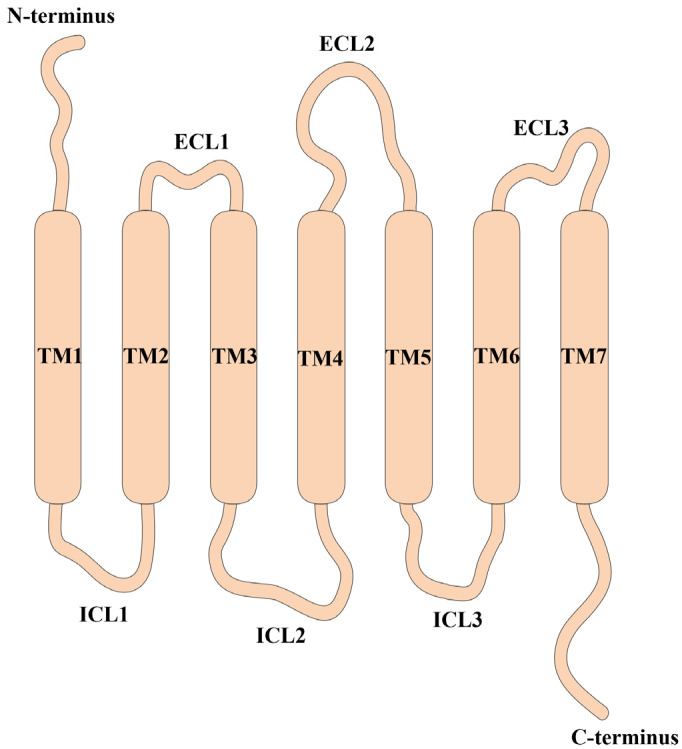
General architecture of CXCRs. CXCRs are characterized by their seven-transmembrane architecture; they have an outward-facing N-terminus, an inward-facing C-terminus, and seven domains (TM1–TM7) that are joined by loop regions. The N-terminal domain is located on the extracellular side of the receptor and is involved in ligand recognition and binding. On the other hand, the C-terminal domain is positioned on the intracellular side of the receptor and interacts with intracellular signaling proteins to propagate downstream signals. Furthermore, extracellular loops (ECL1, ECL2, and ECL3) connect the transmembrane domains on the extracellular side of the receptor. On the other hand, intracellular loops (ICL1, ICL2, and ICL3) connect the transmembrane domains on the intracellular side of the receptor. TM: transmembrane; ECL: extracellular loop; ICL: intracellular loop.

CXCRs are characterized by their seven-transmembrane architecture; they have an outward-facing N-terminus, an inward-facing C-terminus, and seven domains (TM1-TM7) that are joined by loop regions, and this structure forms an active site within the receptor and facilitates the function of CXCRs as cell surface receptors (Figure 1). The alpha-helical segments that form the seven transmembrane domains are responsible for maintaining the structure and stability of the receptor. On the one hand, the N-terminal domain is located on the extracellular side of the receptor and is involved in ligand recognition and binding. On the other hand, the C-terminal domain is positioned on the intracellular side of the receptor and is implicated in regulating receptor internalization, desensitization, and recycling; this domain interacts with intracellular signaling proteins to propagate downstream signals. Furthermore, three loops are present in both the extracellular and intracellular regions of these receptors and perform essential functions in relaying signals across the extracellular and intracellular domains of the receptor. On the one hand, extracellular loops (ECL1, ECL2, and ECL3) connect the transmembrane domains on the extracellular side of the receptor. They participate in ligand binding and contribute to the specificity and affinity of ligand recognition. On the other hand, intracellular loops (ICL1, ICL2, and ICL3) connect the transmembrane domains on the intracellular side of the receptor. As a result, they interact with intracellular signaling molecules and G proteins to initiate downstream signaling events.

To date, seven established human CXCRs, referred to as CXCR1, CXCR2, CXCR3, CXCR4, CXCR5, CXCR6, and CXCR7, have been identified and characterized. Upon the binding of CXCL1, CXCL6, or CXCL8 to CXCR1, neutrophils are recruited to sites of inflammation or infection through a process called chemotaxis [19]. One of these receptors, CXCR2, is primarily associated with the recognition and binding of chemokines such as CXCL1, CXCL3, CXCL5, CXCL7, CXCL2, CXCL6, and CXCL8 and is detected in multiple cell types, including endothelial cells, neutrophils, monocytes, and macrophages. The activation of CXCR2 on these cells can contribute to immune cell recruitment and activation, promote inflammation, and aid in the clearance of pathogens [20]. CXCR3 is activated by specific chemokines, namely, CXCL9, CXCL10, and CXCL11, which are collectively known as IP10, MIG, and I-TAC, respectively, and are produced in response to interferon-gamma. CXCR3 can influence the polarization of T helper (Th) cells toward the Th1 phenotype, allowing them to migrate to and accumulate at sites of inflammation [21,22]. The primary ligand for CXCR4 is CXCL12, which is also known as SDF-1; this protein is secreted by stromal cells that reside in the bone marrow and other tissues and is integral to immune cell development, trafficking, and homing [23,24]. CXCR5 can be triggered or activated by its specific ligand CXCL13, also known as B-lymphocyte chemoattractant (BLC). Upon interaction, CXCL13 engages with CXCR5 receptors on the surface of a subset of T cells named follicular helper T cells (Tfh ) and B cells and initiates a series of signaling events within the cells, leading to a range of functional responses [25,26]. CXCR6 is triggered or activated by its specific ligand CXCL16 and is expressed on various cell types, such as macrophages, dendritic cells, and endothelial cells. The binding of CXCL16 to CXCR6 plays an important role in immune cell migration, adhesion, activation, and tissue-specific immune responses [6,27]. *CXCR7* is activated by its ligands *CXCL11* and *CXCL12* and is expressed by various types of cells, including immune cells, such as T cells, B cells, and dendritic cells, and nonimmune cells such as fibroblasts and endothelial cells. Upon activation, CXCR7 initiates intracellular signaling pathways that regulate various cellular processes, including cell migration, survival, and angiogenesis [8,28]. Consequently, CXCRs have been implicated in various diseases; for example, the CXCR3 and CXCR5 receptors have been implicated in autoimmune conditions, such as multiple sclerosis (MS) [24], rheumatoid arthritis (RA) [29,30], and cutaneous lupus erythematosus (CLE) [31,32]. In addition, CXCR3 is associated with T-cell recruitment and activation in allergic conditions [33]. It has been reported that CXCR2 and CXCR3 are involved in tumor growth and angiogenesis [4,34]. Furthermore, CXCR4 is a receptor by which HIV enters host cells [35]. Overall, CXCRs have the potential to serve as therapeutic targets in cancer [36], inflammation [7], autoimmunity [29,30], fibrosis [37], and allergic diseases [33].

### 2.2. Transmissive Pathways of CXCR Signaling

**Figure 2:**
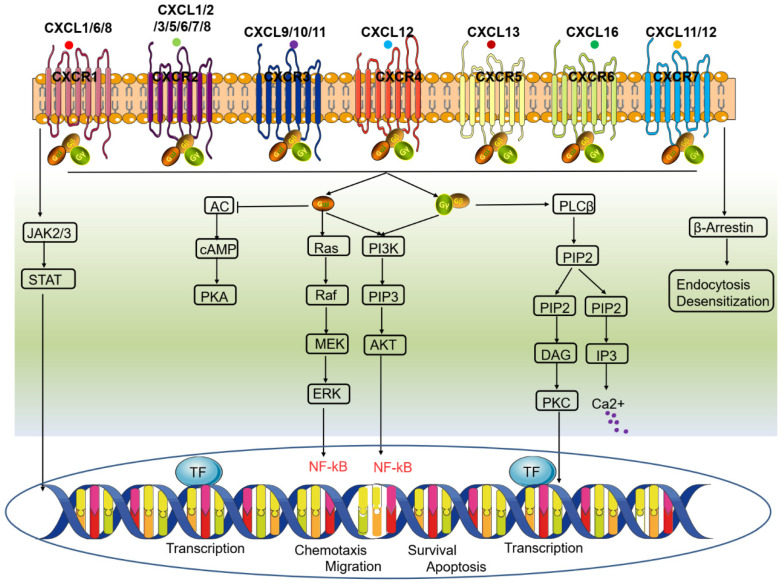
CXCR signaling pathway. Activation of CXCR signaling begins with the binding of ligands to their receptors, triggering conformational changes that activate the receptors. Ligand binding induces interactions between activated CXCR and heterotrimeric G proteins, predominantly Gαi proteins. This interaction leads to the dissociation of Gαi subunits from Gβγ subunits. The activated Gαi subunits and Gβγ subunits subsequently initiate intracellular signaling cascades through various downstream effectors. The Gαi subunit inhibits AC, reducing cAMP levels and subsequently decreasing PKA activity. The Gβγ subunits directly bind to and stimulate PLC-β, which cleaves PIP2 to produce IP3 and DAG. IP3 promotes the release of Ca^2+^ from intracellular stores, while DAG activates PKC. Activated CXCR can also stimulate PI3K, leading to the synthesis of PIP3. PIP3 recruits and activates Akt, which regulates cell survival, proliferation, and other cellular processes. CXCR activation initiates the activation of MAPK cascades, including the JNK, p38 MAPK, and ERK pathways, which are involved in cell proliferation, differentiation, and gene expression regulation. Additionally, CXCR activation triggers the activation of NF-κB, which is a transcription factor that regulates inflammatory and immune responses. CXCR: C-X-C motif chemokine receptor; AC: adenylyl cyclase; cAMP: cyclic adenosine monophosphate; PKA: protein kinase A; PLC-β: phospholipase C-β; PIP2: phosphatidylinositol 4,5-bisphosphate; IP3: inositol trisphosphate; DAG: diacylglycerol; Ca^2+^: calcium ions; PKC: protein kinase C; PI3K: phosphatidylinositol 3-kinase; PIP3: phosphatidylinositol (3,4,5)-trisphosphate; Akt: protein kinase B; MAPK: mitogen-activated protein kinase; JNK: c-Jun N-terminal kinase; ERK: extracellular signal-regulated kinase; NF-κB: nuclear factor kappa-B.

Activation of CXCR signaling typically occurs when a ligand binds to the corresponding receptor, which induces conformational changes in the receptor, leading to its activation. Upon ligand binding, activated CXCR proteins interact with heterotrimeric G proteins, primarily Gαi proteins (Figure 2). As a result, these interactions cause the dissociation of Gαi subunits from Gβγ subunits, after which the activated Gαi subunits and Gβγ subunits initiate intracellular signaling cascades through a range of downstream effectors. On the one hand, Gαi subunits can inhibit adenylyl cyclase (AC), reducing cyclic adenosine monophosphate (cAMP) levels and leading to decreased protein kinase A (PKA) activity. Conversely, Gβγ subunits can directly bind to and stimulate phospholipase C-β (PLC-β), triggering the enzymatic cleavage of phosphatidylinositol 4,5-bisphosphate (PIP2) and leading to the synthesis of inositol trisphosphate (IP3) and diacylglycerol (DAG). In addition, IP3 prompts the discharge of calcium ions (Ca^2+^) from intracellular pools, whereas protein kinase C (PKC) is activated by DAG. Activated CXCR can stimulate PI3K, resulting in the synthesis of phosphatidylinositol (3,4,5)-trisphosphate (PIP3). Thus, Akt (protein kinase B) is recruited and activated and can regulate cell survival, proliferation, and other cellular processes. In addition, upon CXCR activation, mitogen-activated protein kinase (MAPK) cascades, such as the c-Jun N-terminal kinase (JNK), p38 MAPK, and extracellular signal-regulated kinase (ERK) pathways, which are associated with cell proliferation, differentiation, and gene expression regulation, can also be initiated. Additionally, activation of CXCR can activate nuclear factor kappa-B (NF-κB), a transcription factor involved in regulating inflammatory and immune responses. Notably, the specific signaling pathway and cellular responses can differ depending on the specific CXCR subtype (e.g., CXCR2, CXCR4) and the cellular context in which CXCR is activated.

## 3. CXCRs in Dermatological Inflammatory Conditions

**Figure 3:**
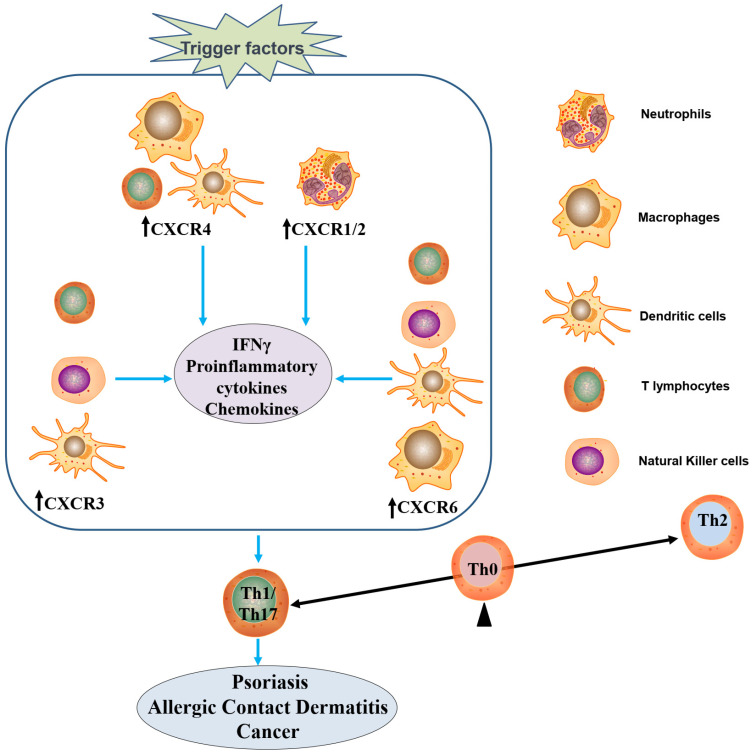
Proposed conceptual framework for the involvement of CXCR signaling in modulating Th1/Th17-driven pathologies. Initiation of Th1/Th17 T-cell development can be triggered by the activation of multiple CXCRs, including CXCR1 and CXCR2 in neutrophils (NEUs); CXCR3 in dendritic cells (DCs), natural killer cells (NKs), and T lymphocytes (T cells); CXCR4 in DCs, T cells, and macrophages (Mφ); and CXCR6 in DCs, T cells, Mφ, and NKs. Activation of these CXCR-mediated signaling cascades triggers upregulated secretion of proinflammatory cytokines, type II interferons (predominantly IFNγ produced by T cells), and chemokines, which eventually facilitate the acquisition of the Th1/Th17 phenotype by T cells, progressing from the Th0 stage. Activation of the Th1/Th17 phenotype supports the recruitment and activation of immune cells, enabling a robust immune response against both pathogens and environmental insults, but hyperstimulation of the Th1/Th17 pathway is associated with the pathogenic processes of multiple skin disorders, including allergic contact dermatitis, psoriasis, and cancers.

CXCRs are expressed in many skin cells and play critical roles in skin physiology and pathophysiology (Figure 3). The balanced expression and function of CXCRs in a range of skin cell types are essential for maintaining skin homeostasis. Dysregulation or dysfunction of CXCR signaling can disrupt balance and lead to skin disorders, such as impaired barrier function, abnormal cell migration, immune dysregulation, pigmentation disorders, and vascular abnormalities. Understanding the roles of CXCRs in skin homeostasis provides insights into the mechanisms underlying human health and contributes to the development of potential therapeutic strategies for managing human diseases [38].

### 3.1. Expression of CXCRs and Functional Implications in Skin Cells

Keratinocytes, which are the predominant cells in the epidermis, express several CXCRs, including *CXCR1*, *CXCR2*, *CXCR4*, and other related receptors, which are involved in attracting a variety of leukocytes, thus contributing to the modulation of inflammatory and immune responses. These receptors are involved in various processes, such as regulating keratinocyte proliferation, migration, and differentiation. For instance, reduced *CXCR2* expression or impaired CXCR2-mediated signaling in keratinocytes can lead to delayed or impaired re-epithelialization and compromise wound closure [39,40]. *CXCR4* expression is increased in atopic dermatitis skin lesions [41], and CXCR4/CXCL12 signaling in keratinocytes contributes to the infiltration of inflammatory cells, such as T cells and eosinophils, into the skin [28].

Fibroblasts, which are found in the dermal layer of the skin, express *CXCR1, CXCR2, CXCR4*, and other related receptors, which play vital roles in maintaining skin homeostasis. On the one hand, activation of these receptors by their respective chemokine ligands can induce fibroblast migration toward the chemokine gradient. CXCR4, in particular, is involved in directing fibroblast migration during wound healing and tissue repair [42,43]. In addition, fibroblasts are also responsible for producing and maintaining the ECM, which provides structural support for tissues. In resolved ACD, T cells influence tissue inflammation and facilitate MMP-12-related tissue modulation [44]. CXCR signaling in fibroblasts can regulate ECM remodeling. For example, CXCR4 activation stimulates the production of MMPs, which are enzymes involved in ECM degradation and turnover [30]. In other words, CXCR signaling can influence fibroblast proliferation and survival. Activation of CXCR1 and CXCR2 can promote fibroblast proliferation, while CXCR4 activation has been shown to enhance fibroblast survival and resistance to apoptosis [45,46]. Moreover, fibroblasts contribute to angiogenesis, which is the formation of new blood vessels. CXCR4 signaling in fibroblasts can promote angiogenesis by stimulating the secretion of angiogenic factors and recruiting endothelial progenitor cells [47,48]. Aberrant CXCR signaling in fibroblasts has been implicated in fibrotic disorders, during which excessive collagen deposition and tissue remodeling occur. The CXCL12/CXCR4 axis presents an incredible challenge and opportunity in the treatment of fibrosis [37]. CXCR4 signaling, in particular, has been associated with the activation and differentiation of fibroblasts into myofibroblasts, which are responsible for excessive ECM production in fibrotic tissues [49].

The involvement of CXCR is crucial for maintaining homeostatic control over T cells that participate in immune surveillance [50]. T-cell subsets play a crucial role in skin physiology and exert marked effects on inflammatory skin disorders [51]. CXCRs are expressed on the cell membranes of T cells and play crucial roles in T-cell migration, activation, and immune responses. High levels of *CXCR3* expression are observed in activated T cells, and effector and memory T cells express notable levels of this receptor [52]. The interaction between CXCR3 and its ligands CXCL9, CXCL10, and CXCL11 is facilitated by inflammatory stimuli [21,22]. CXCR3 signaling is involved in modulating the recruitment of T cells to sites of inflammation, including sites of infection or autoimmune inflammation. Tregs and CD4+ and CD8+ T cells express *CXCR4* on their surfaces. CXCR4 interacts with its ligand CXCL12, which exerts inflammatory effects on other chemokines to induce leukocyte chemotaxis [53]. CXCR4 signaling regulates T-cell trafficking and homing to lymphoid organs, bone marrow, and inflamed tissues. *CXCR5* is expressed on T cells that belong to the Tfh subset. CXCR5 interacts with its ligand CXCL13, which is produced by follicular dendritic cells in lymphoid follicles. CXCR5 signaling is crucial for Tfh-cell migration to B-cell follicles within lymphoid organs, where they aid in promoting antibody production and affinity maturation in B cells. *CXCR6* is expressed on multiple T-cell subsets, namely, effector/memory CD4+ and CD8+ T cells. Its ligand, CXCL16, is detected on antigen-presenting cells, including macrophages and dendritic cells. CXCR6 signaling regulates T-cell migration to inflamed tissues, namely, the gut, kidney, and liver. Thus, the function of CXCR is essential for modulating the stability and homeostasis of T cells that participate in immune surveillance.

### 3.2. Activation of CXCRs and the Onset of ACD

ACD is a delayed hypersensitivity response that occurs upon the exposure of skin to a particular substance (known as an allergen) to which the individual is sensitized [54]. ACD ranks among the most prevalent chronic inflammatory skin conditions, affecting approximately 20% of people worldwide [55], and has a similar prevalence in both pediatric and adult populations [56]. The principal histological features of ACD are spongiosis, acanthosis, inflammatory infiltration, and edema. Currently, there is no cure for ACD, and only treatments that alleviate symptoms are available [57]. The factors that trigger the recurrence of ACD can vary among individuals, but there are some common factors that can contribute to flare-ups, such as allergens (metals such as nickel [58] and cobalt, fragrances, preservatives, latex), irritants (harsh soaps, detergents, solvents, or other chemicals) [59,60,61,62], and skin barrier disruption. However, due to their persistent sensitization, patients with ACD often experience a high rate of recurrence [63].

ACD is considered a T-cell-mediated disease because it involves a specific immune response that is mediated by T lymphocytes [57]. Briefly, ACD development includes two stages, namely, the sensitization and elicitation phases. In the sensitization phase, allergen-specific T lymphocytes are produced, which subsequently induce skin inflammation upon exposure to the same antigen. In the elicitation phase, allergen-specific T lymphocytes migrate to the inflammatory site and elicit a reaction against the antigen [64]. Differential chemokine responses play crucial roles in distinguishing between chemical-induced allergic and irritant skin inflammation, with memory T cells being the differentiating factor [65]. Numerous studies have indicated that T cells contribute to inflammatory skin conditions, such as ACD, primarily through the production of Th1 or Th17 cytokines [66].

Cytokine receptors are required for the maintenance and survival of pathogenic T cells during and after ACD reactions. The accumulation of CD8+ and CD4+ T cells in inflamed skin is controlled by distinct chemokines and cell populations [15]. Neutrophil recruitment is orchestrated by CD8+ tissue-resident memory T cells [67], which promote the production of CXCL1 and CXCL2 in ACD-affected skin. Furthermore, blocking CXCR1 and CXCR2 effectively suppresses flare-up reactions and prevents neutrophil infiltration [68]. It is essential to attenuate excessive neutrophil activation and infiltration to protect the host from harm [69]. In ACD, specific chemokine signaling pathways are selectively activated, and CXCR3 and its endogenous chemokines CXCL9, CXCL10, and CXCL11 play prominent roles in activating unique intracellular signaling cascades [14]. These distinct signaling patterns present an appealing therapeutic target for the development of novel treatments for ACD and other inflammatory conditions. Notably, the chemokine receptor CXCR3 serves as a key regulator of inflammation in different diseases by facilitating the migration of effector/memory T cells. However, a previous study highlighted that CXCR3-targeted agonists elicit distinct physiological responses. Selective agonists of the chemokine receptor CXCR3 exert varying regulatory effects on chemotaxis and inflammation, which warrants careful consideration for future research endeavors [70].

In summary, ACD is a prevalent dermatological condition that is characterized by inflammation and irritation of the skin due to exposure to certain substances called allergens. CXCR-expressing cells, such as T lymphocytes and other inflammatory cells, are attracted by cytokines and migrate to the site of skin damage [23]. Modulating T-cell activation can help alleviate symptoms and reduce the severity of ACD. However, the main problem associated with targeting T-cell activation in the treatment of ACD is the balance between effective suppression of the immune response and potential adverse effects. In the future, the development and use of CXCR-targeted therapies are highly promising for the management of ACD.

### 3.3. Activation of CXCRs and Initiation of Psoriatic Inflammation

The hallmark features of psoriasis include chronic inflammation, autoimmune dysregulation, and rapid skin cell proliferation, which result in the formation of thick, scaly patches on the skin [71]. Approximately 2–3% of people worldwide are believed to be affected by psoriasis, which translates to approximately 125–200 million people worldwide. Psoriasis is characterized by distinct histological features, such as a thickened epidermis, parakeratosis, psoriasiform hyperplasia, Munro’s microabscess, inflammatory cell infiltration, and dilated blood vessels. Psoriasis is a chronic condition, and there is currently no known cure [72]. The factors that trigger psoriasis recurrence vary among individuals, but several recurring factors, such as stress, skin injury, infection, medication (beta-blockers, lithium salts, and nonsteroidal anti-inflammatory drugs), smoking, and alcohol consumption, can often trigger or worsen psoriasis flare-ups [73,74].

Psoriasis is considered a T-cell-mediated disease because abnormal activation of T cells, specifically subsets called Th1 cells and Th17 cells, is implicated in the pathogenesis of this disease [75,76,77]. These activated T cells produce a range of proinflammatory cytokines, namely, TNF-α, IL-17, and IL-23 [78,79,80]. As these cytokines are released, they enhance the inflammatory process, playing a role in the development of the hallmark features of psoriasis, including increased proliferation of skin cells and recruitment of other immune cells. Once activated, keratinocytes secrete a range of proinflammatory cytokines, such as TNF-α, IL-17, IL-23, IL-1, IL-6, IL-22, IL-8, and IL-12 [81], and chemotactic cytokines, such as CXCL8, CCL20, CXCL9, CXCL10, CCL17, and CCL27, that contribute to the development and progression of psoriatic lesions [81,82,83]. Once produced, chemokines are released by keratinocytes into the surrounding tissue or the bloodstream [84]. They can diffuse through the extracellular matrix or be transported by other cells, such as neutrophils or dendritic cells [69], to reach their target locations and bind to chemokine receptors. In psoriatic skin, *CXCR4* expression is increased specifically in the junctional region adjacent to psoriatic plaques. Unexpectedly, CXCR4 plays a role in suppressing KC proliferation and mitigating the impact of proliferative T helper 17 cytokines [11]. In psoriasis, CXCL16 and CXCR6 are elevated and are involved in recruiting human CD8+ T cells to the skin [12]. Thus, the recruitment and activation of immune cells through the CXCL–CXCR cascade enhance the inflammatory response in psoriasis.

In psoriasis, abnormal and excessive skin cell proliferation occurs, and it is driven by an autoimmune process in which the immune system mistakenly targets healthy skin cells, eliciting an aberrant immune response [85]. These immune cells, which express *CXCR*, interact with chemokines and their respective receptors to regulate their migration, positioning, and activation within psoriatic lesions. These events are strongly associated with T-cell activation and the development and progression of the disease. Strategies that specifically target T-cell activation and the expression of proinflammatory cytokines have proven to be effective approaches for the treatment of psoriasis. Medications, such as biologics and small molecules, can modulate T-cell activity and cytokine signaling to reduce inflammation and improve symptoms. However, the most significant potential problem associated with targeting T-cell activation in psoriasis is the risk of immunosuppression. While suppressing T-cell activation can effectively reduce inflammation and alleviate psoriasis symptoms, it also compromises the ability of the immune system to defend against infections and diseases. This increased susceptibility to infections is a major concern when using T-cell-targeted therapies. In the future, the development and use of CXCR-targeted therapies hold great potential in the management of psoriasis.

## 4. CXCR-Specific Therapies

CXCR is involved in chemotaxis, immune cell activation, and inflammation regulation and plays a crucial role in immune responses and maintaining tissue homeostasis. Abnormalities in CXCRs contribute to the development of multiple inflammatory skin conditions; for this reason, targeting CXCRs can potentially provide therapeutic benefits for conditions, such as atopic dermatitis, psoriasis, and other inflammatory skin disorders. CXCR modulators, including agonists and antagonists, have been approved or are currently in development for the treatment of skin diseases. We will next review the significance of CXCR in therapy, recent advances, and future outlooks in managing skin diseases (Table 1).

### 4.1. Application of CXCR as a Therapeutic Target in Allergic Contact Dermatitis

The limitations of traditional therapies for ACD can be explained by several factors. First, these therapies often focus on providing symptomatic relief rather than addressing the underlying immune response and the specific signaling pathways involved. Second, traditional treatments may not effectively target key mediators and signaling molecules that are responsible for allergic reactions, leading to incomplete suppression of inflammation and persistent symptoms. Additionally, the chronic nature of ACD may require long-term treatment, and traditional therapies may not offer optimal control or prevention of flare-ups. Finally, individual variations in immune responses and the diverse range of allergens involved can further contribute to the limitations of traditional therapies in providing consistent and tailored treatment approaches.

Here, we provide a few examples of clinical and research progress in the development of therapies that target CXCRs. Researchers are exploring the use of CXCR3 antagonists, such as AMG487 [86] and NBI74330 [87], that specifically block CXCR3 signaling and inhibit the migration of inflammatory cells to affected skin. Preclinical studies have demonstrated encouraging outcomes for these antagonists, and they are currently being evaluated in clinical trials. A range of CXCR2 antagonists, such as SCH527123 [88] and SB-656933 [89], have been developed, and their potential to reduce inflammation and symptoms is being investigated. Clinical studies are underway to evaluate the safety and efficacy of these agents. Ulocuplumab and plerixafor, which are monoclonal antibodies that target CXCR4 [90,91], have been studied due to their ability to block CXCR4 signaling and inhibit immune cell migration. These antibodies hold promise as potential therapeutic options for ACD and are currently being assessed in clinical trials. Given the involvement of both CXCR3 and CXCR4 in ACD, researchers are also exploring the development of dual antagonists that simultaneously target both receptors. These dual antagonists aim to provide broader inhibition of inflammatory cell recruitment and potentially offer enhanced therapeutic benefits. Several dual CXCR3/CXCR4 antagonists, such as LY2510924 [90] and CCX771 [92], are being investigated in preclinical and early clinical studies of inflammatory diseases. These examples highlight the ongoing research and efforts to develop drugs that target CXCR and modulate the immune response in ACD patients. While these therapies are still under investigation, they hold promise for potentially more targeted and effective treatment options in the future.

### 4.2. Prospect of CXCR Inhibition as a Therapeutic Approach for Psoriasis

The limitations of traditional therapies for psoriasis are explained by several factors. First, these therapies primarily focus on managing symptoms rather than addressing the underlying immune dysregulation that drives the disease. Second, the side effects associated with some traditional treatments, such as corticosteroids, can pose risks when administered over long periods or at high doses. Additionally, the efficacy of traditional therapies can vary among individuals, with some patients experiencing minimal improvement and some experiencing no response at all. Last, the chronic nature of psoriasis often leads to relapses after traditional treatments are discontinued. The limitations of traditional psoriasis treatments can be attributed to their inability to specifically target key signaling pathways that are involved in the pathogenesis of the disease. Here, we provide a few examples of signaling pathways that play a role in psoriasis and are not effectively targeted by traditional therapies.

There are several clinical or investigational approaches for targeting CXCRs. LY2510924, which is a monoclonal antibody that targets CXCR4, has been investigated in clinical trials as a potential treatment for a variety of diseases [91]. BL8040, which is a CXCR4 antagonist, is being evaluated in clinical trials as a potential therapy for cancer [93]. MDX1100, which is a monoclonal antibody that targets CXCL10, is a CXCR3 ligand that has been investigated in clinical trials as a potential therapy for arthritis [94]. These examples represent some of the clinical or investigational approaches for targeting CXCRs or their ligands in the treatment of psoriasis. Importantly, the efficacy and safety of these treatments are still under investigation, and additional research is needed to determine their overall effectiveness in the management of psoriasis.

### 4.3. Therapeutic Use of CXCR Ligands for Treating Skin Disorders and Cancer

The therapeutic application of CXCR ligands involves utilizing these molecules to target CXCRs and modulate immune responses and inflammation. In malignancies (e.g., lymphoma and multiple myeloma), cancer cells can evade the immune system and reside in the bone marrow, hindering effective treatment. AMD3100, which is an SDF-1α/CXCR4 inhibitor approved by the FDA, has been shown to mitigate radiation-induced skin damage and fibrosis [95] and is used to treat hematopoietic stem cell mobilization for stem cell transplantation in patients with certain types of cancer [96,97]. By selectively binding to CXCR, these ligands can regulate immune cell migration and function, offering potential benefits in the treatment of various diseases, including skin disorders, autoimmune diseases, and cancer.

## 5. Conclusions

The skin, which functions as a primary barrier, is continually subjected to pathogenic or hazardous elements in the surrounding environment. CXCRs, which are integral molecules that promote chemotaxis, contribute to the initiation of immune cell migration, activation of immune cells, modulation of adaptive immune responses, and ultimately, the generation of both immediate and long-lasting immune protection. The available evidence is increasingly suggesting that CXCRs play critical roles in the development of various inflammatory skin conditions. As a result, therapeutic interventions that target CXCRs have been designed and explored, with the aim of suppressing the dysregulated activation of CXCRs that contributes to autoimmune activation and reducing the side effects on noninflammatory cells. Selecting interventions that specifically target effector cells at later stages of disease progression may offer improved therapeutic benefits. With recent achievements in targeting T cells and effector cells in later stages of disease progression, therapeutic strategies that involve targeting both T cells and CXCR signaling during disease progression have become increasingly preferred. Nonetheless, the most significant limitation of targeting CXCR proteins involves the development of drug resistance. However, the development of drug resistance is a common issue, not only in the context of targeting CXCRs but also in other targeted treatment approaches. Ongoing studies are needed to devise strategies that combine CXCR-targeted therapy with other treatment modalities, such as phototherapy or immunotherapy, and to explore individualized CXCR-targeted treatment approaches based on patients’ genotypic characteristics; these approaches can be utilized to establish more enduring treatments for inflammatory skin conditions, such as allergic contact dermatitis or psoriasis.

## Figures and Tables

**Table 1:** Summarized applications of CXCR as a therapeutic target.

Biological Drugs	Target sites	Categories	Therapeutic Applications	References
AMG487	CXCR3	Antagonist	Block CXCR3 signaling and inhibit the migration of inflammatory cells	[86]
NBI74330	CXCR3	Antagonist	Block CXCR3 signaling and inhibit the migration of inflammatory cells	[87]
SCH527123	CXCR2	Antagonist	Reduce inflammation and symptoms	[88]
SB656933	CXCR2	Antagonist	Reduce inflammation and symptoms	[89]
Ulocuplumab	CXCR4	Monoclonal antibody	Block CXCR4 signaling and inhibit immune cell migration	[90,91]
Plerixafor	CXCR4	Monoclonal antibody	Block CXCR4 signaling and inhibit immune cell migration	[90,91]
LY2510924	Dual CXCR3 /CXCR4	Antagonists	Clinical studies of inflammatory diseases	[90]
CCX771	Dual CXCR3 /CXCR4	Antagonists	Clinical studies of inflammatory diseases	[92]
LY2510924	CXCR4	Monoclonal antibody	A potential treatment for inflammatory diseases	[91]
BL8040	CXCR4	Antagonist	A potential therapy for cancer	[93]
MDX1100	CXCL10	Monoclonal antibody	A potential therapy for arthritis	[94]
AMD3100	CXCR4	Inhibitor	Radiation skin damage and fibrosis reduction, hematopoietic stem cell mobilization for cancer patients’ transplantation	[95]

## Data Availability

Not applicable.
